# Hemoglobinuria-related acute kidney injury is driven by intrarenal oxidative reactions triggering a heme toxicity response

**DOI:** 10.1038/cddis.2015.392

**Published:** 2016-01-21

**Authors:** J W Deuel, C A Schaer, F S Boretti, L Opitz, I Garcia-Rubio, J H Baek, D R Spahn, P W Buehler, D J Schaer

**Affiliations:** 1 Division of Internal Medicine, University of Zürich, Zürich, Switzerland; 2 Institute of Anesthesiology, University of Zürich, Zürich, Switzerland; 3 Clinic for Small Animal Internal Medicine, University of Zürich, Zürich, Switzerland; 4 Functional Genomics Center Zürich, Swiss Federal Institute of Technology Zürich/University of Zürich, Zürich, Switzerland; 5 Laboratory of Physical Chemistry, ETH Zürich, Zürich, Switzerland; 6 Centro Universitario de la Defensa, Carretera de Huesca, Zaragoza, Spain; 7 Laboratory of Biochemistry and Vascular Biology, Center of Biologics Evaluation and Research (CBER), FDA, Silver Spring, MA, USA; 8 Institute of Evolutionary Medicine, University of Zürich, Zürich, Switzerland

## Abstract

Intravascular hemolysis can result in hemoglobinuria with acute kidney injury. In this study we systematically explored two *in vivo* animal models and a related cell culture system to identify hemoglobinuria-triggered damage pathways. In models of stored blood transfusion and hemoglobin (Hb) exposure in guinea pigs and beagle dogs we found that hemoglobinuria led to intrarenal conversion of ferrous Hb(Fe^2+^) to ferric Hb(Fe^3+^), accumulation of free heme and Hb-cross-linking products, enhanced 4-hydroxynonenal reactivity in renal tissue, and acute tubule injury. These changes were associated in guinea pigs with activation of a renal cortex gene expression signature indicative of oxidative stress and activation of the unfolded protein response (UPR). Tubule cells of hemolytic animals demonstrated enhanced protein expression of heme oxygenase and heat shock protein and enhanced expression of acute kidney injury-related neutrophil gelatinase-associated lipocalin. These adverse changes were completely prevented by haptoglobin treatment. The *in vivo* findings were extrapolated to a MS-based proteome analysis of SILAC-labeled renal epithelial cells that were exposed to free heme within a concentration range estimate of renal tubule heme exposure. These experiments confirmed that free heme is a likely trigger of tubule barrier deregulation and oxidative cell damage and reinforced the hypothesis that uncontrolled free heme could trigger the UPR as an important pathway of renal injury during hemoglobinuria.

Hemolysis is a common pathophysiologic process. It occurs in numerous conditions, including genetic hemoglobinopathies and malaria, the transfusion of stored red blood cells, and during therapeutic procedures requiring extracorporeal assist. These conditions may release hemoglobin (Hb), which contributes to hemolysis-associated adverse clinical outcomes, such as endothelial dysfunction, oxidative vascular toxicity, and kidney injury.^1^

The toxicity of cell-free Hb has been attributed to a number of its unique properties. First, Hb readily decays into heterodimers that are considered to be small enough to extravasate and may enter tissues, such as the kidney, that have less antioxidant capacity than blood.^1^ Second, Hb interacts with ligands other than oxygen, namely nitric oxide (NO) and peroxides.^2, 3^ These reactions are related to NO depletion and vascular dysfunction and may trigger oxidative tissue damage during free Hb exposure. Third, ferric Hb(Fe^3+^), which is a product of Hb autoxidation or Hb reactions with endogenous oxidants, can release free heme. Free heme is a potent trigger of lipid peroxidation and a promoter of inflammation.^4, 5, 6^ At high concentrations, heme can act as an endogenous inhibitor of the proteasome and as a trigger of the response to unfolded proteins *in vitro*.^7^

Under physiologic conditions, Hb toxicity remains controlled by a specialized scavenger system. Free Hb is bound by the plasma protein haptoglobin, and the large molecular size Hb:Hp complexes are ultimately cleared by spleen and liver macrophages expressing the Hb scavenger receptor CD163.^8^ However, during more severe hemolysis these protective systems are saturated and glomerular filtration of free Hb becomes the major pathway that removes Hb from the circulation. In a recent pharmacokinetic analysis of free Hb clearance in dogs, we determined that free Hb in plasma is cleared from the circulation with a half-life of <1 h.^9^ Although the renal clearance of Hb is a fast and very effective way to limit the Hb exposure of other critical tissues such as the vasculature, the kidney itself can be exposed to large quantities of free Hb. Therefore, acute kidney injury (AKI) remains an important complication of acute and severe intravascular hemolysis. A paradigmatic example is Hb-triggered AKI as a complication of severe malaria with hemoglobinuria. This condition was called black water fever owing to its impressive clinical presentation with dark-colored urine.^10^ Renal damage has also been reported to occur throughout many other acute hemolytic conditions associated with hemoglobinuria.^11, 12, 13, 14, 15, 16, 17, 18^ The mechanisms of renal damage during these conditions are considered to be multifactorial and may involve direct damage to renal tubule cells by glomerular-filtered Hb.^18^ However, no systematic studies have so far been reported to explore the molecular mechanisms of Hb-triggered renal damage during hemolysis.

In the current study, we searched for hemoglobinuria-associated changes of the renal cortical tissue transcriptome. In addition, we investigated disease-related biochemical and molecular pathways that may be activated in heme-exposed renal epithelial cells. Cumulatively, our findings show that renal Hb exposure led to the accumulation of large quantities of oxidized ferric Hb(Fe^3+^) in tissues, heme release, and secondary radical reactions. These conditions may promote a heme-related cellular damage pathway in tubule epithelial cells that is characterized by an oxidative stress response and an activated response to unfolded proteins, which may ultimately lead to renal tubule toxicity and AKI.

## Results

### The renal transcriptional response to hemoglobinuria in guinea pigs indicates that tubular damage is caused by oxidative stress and an activated response to unfolded proteins

Isovolemic transfusion of guinea pigs with 10-unit equivalents of old blood, which consisted of red blood cells that were stored for 28 days, led to moderate hemoglobinuria.^14^ Old blood transfusion resulted in systemic free Hb total exposure (AUC_0–∞_) of 4600 *μ*mol heme-h/l, which was primarily cleared by the kidney within 24 h. No measurable hemolysis occurred after the transfusion of 10-unit equivalents of new blood.

**Figure 1:**
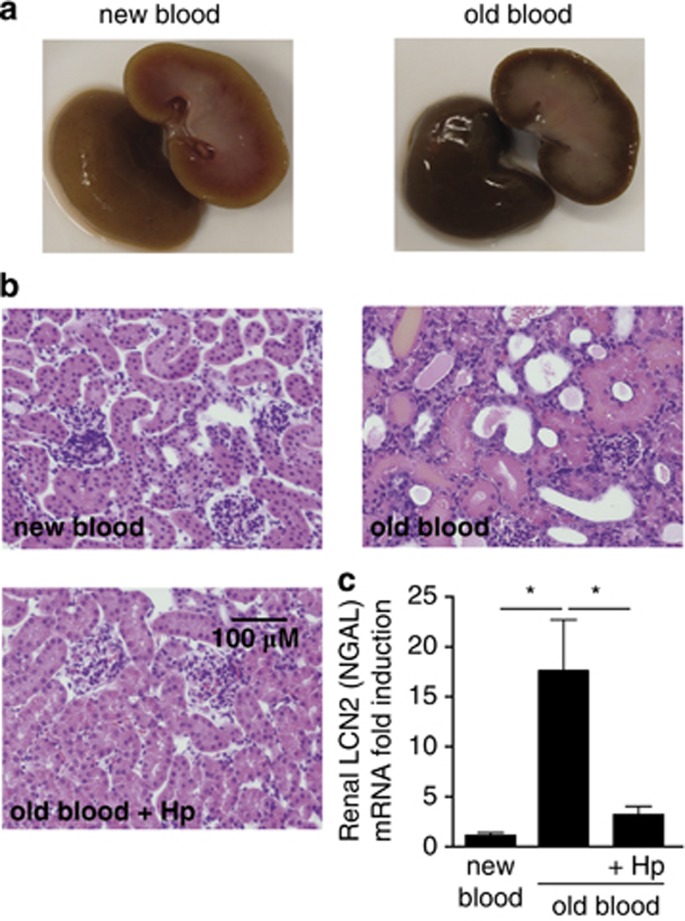
Renal outcome of blood transfusion-related hemolysis in guinea pigs. (**a**) Macroscopic view of kidneys of new blood-transfused and old blood-transfused guinea pigs at 24 h after treatment. (**b**) Hematoxylin and eosin-stained kidney sections of new blood-transfused, old blood-transfused, and old blood-transfused, haptoglobin (Hp)-treated animals at 24 h after treatment. (**c**) Renal tissue expression of lipocalin 2 (LCN2)/neutrophil gelatinase-associated lipocalin (NGAL) mRNA at 24 h after treatment (*P<0.01)

The transfusion-associated hemolysis resulted in brown discoloration of the kidney, swelling of tubule epithelial cells, and formation of eosinophil casts in the tubular lumen (Figure 1). These changes were associated with an increase in the mRNA expression of neutrophil gelatinase-associated lipocalin (NGAL), indicating AKI with tubular cell damage.^19^ No changes in the morphology or NGAL expression were observed after the transfusion of fresh blood. Hp treatment at the time of the transfusion of the old blood prevented the glomerular filtration of Hb as well as changes in morphology or NGAL expression after the transfusion.

**Figure 2:**
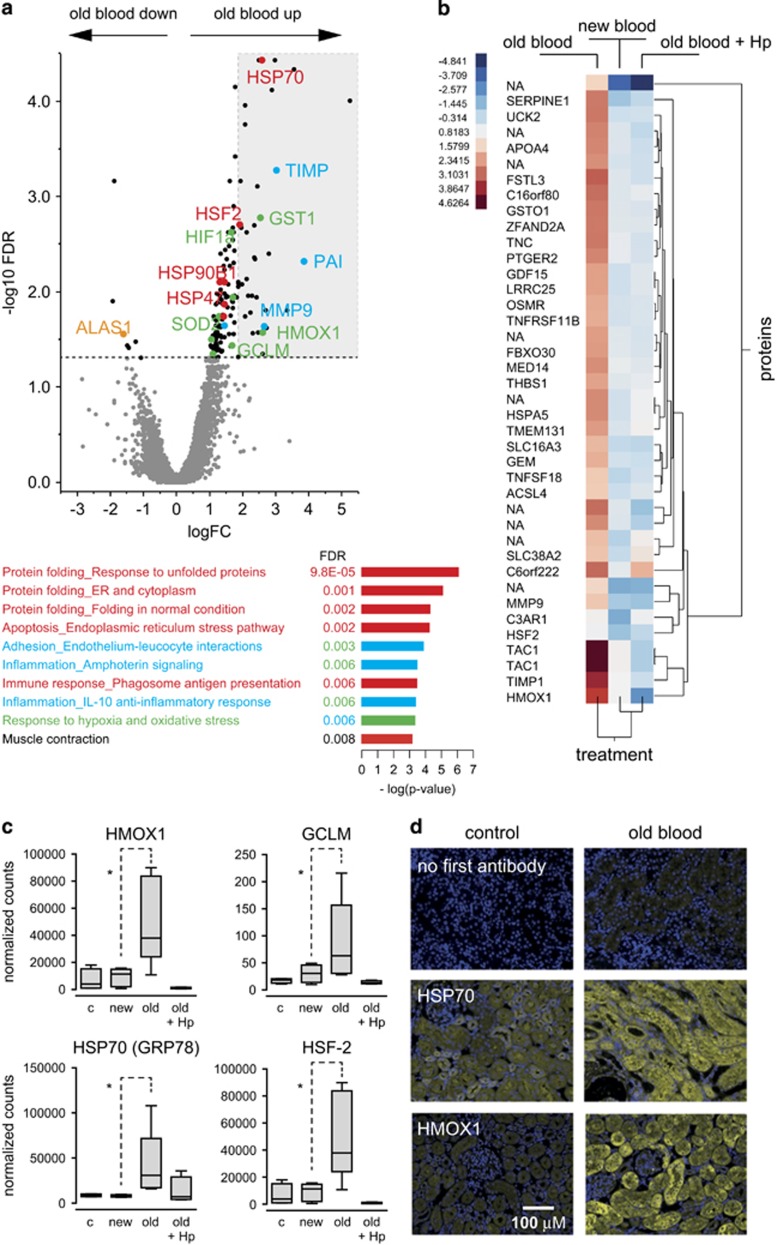
Hemolysis triggered renal gene expression. (**a**) Volcano plot of all gene expression tags that were found by serial analysis of gene expression (SAGE) of guinea pig renal tissues. LogFC indicates the relative gene expression in renal cortical tissues of old blood-transfused *versus* new blood-transfused animals. The threshold of significance was set at a false discovery ratio (FDR)<0.05. Selected transcripts are labeled and color-coded for related functions: red—protein folding/response to unfolded proteins; green—response to oxidative stress; blue—inflammation. The significant results of a MetaCore analysis for the enrichment of functional networks among significantly upregulated genes are shown at the bottom. The light-gray area in the volcano plot highlights the top 40 upregulated genes that were re-analyzed in the hierarchical clustering analysis shown in (**b**). The color-coded gene expression indicates the ratio of normalized SAGE-tagged counts of treated *versus* untreated control tissues (upregulated genes are red). (**c**) Box plots of SAGE mRNA transcript counts for selected genes (*P<0.05). (**c**) Sample box plots of normalized sequence counts. *p<0.05. (**d**) Immunofluorescence analysis of heat shock protein 70 (Hsp70) and heme oxygenase-1 (HMOX1) in renal tissues of control and old blood-transfused animals. Control slices were stained with goat anti-rabbit (Alexa 568) secondary antibody without any primary antibody. Images were acquired with a Zeiss Axioskop 2 epifluorescence microscope at an original magnification of × 200

We obtained renal gene expression profiles from these kidneys using serial analysis of gene expression (SAGE). We analyzed renal cortical tissues that were collected from control (*n*=4), new blood-transfused (*n*=6), old blood-transfused (*n*=5), and Hp-treated, old blood-transfused (*n*=4) animals. At a false discovery rate (FDR) of 5%, we found 156 transcripts that were differentially regulated in the old blood-transfused (hemoglobinuria) animals compared with the new blood-transfused (no hemoglobinuria) animals (Figure 2a, and Supplementary data). In total, 149 transcripts were significantly upregulated in the old blood-transfused animals. Along with the suppression of the endogenous heme synthesis pathway (i.e., 5′-aminolevulinate synthase 1), the induction of heme oxygenase-1 (HMOX1) suggests that this response is driven by the exposure to free heme.

A MetaCore analysis of the upregulated genes for significantly enriched functional networks is also shown in Figure 2a. The highest scores are associated with the response to unfolded proteins and the endoplasmic reticulum (ER) stress pathway of apoptosis, as well as the response to oxidative stress. Signature genes representing these processes, as well as inflammation-related transcripts, are color-coded in the volcano plot. Figure 2b shows a hierarchical clustering analysis of the 40 most upregulated genes across all treatment conditions (including new blood transfusion and old blood+Hp transfusion data). The clustering analysis clearly suggests that the Hp-mediated prevention of hemoglobinuria normalizes the renal gene expression pattern toward the renal gene expression profile of new blood-transfused animals. The Hp rescue supports the hypothesis that renal gene expression changes in this model represent a specific renal response to Hb/heme toxicity. Figure 2c shows sample box plots of normalized sequence counts for heat shock factor 2, heat shock protein 70 (HSP70 member GRP78), glutamate–cysteine ligase modifier subunit, and HMOX1.

Immunofluorescence confirmed that renal Hb exposure triggered overexpression of HMOX1 and the unfolded protein response (UPR) chaperone HSP70 in tubule epithelial cells (Figure 2d).

### Hemoglobinuria-associated intrarenal oxidative processes and free heme release

To substantiate the hypothesis that oxidative processes occur in the kidney during acute hemoglobinuria, we examined the renal tubule Hb/iron exposure, ferric Hb(Fe^3+^) accumulation, and markers of oxidative protein modification in the kidneys of old blood-transfused guinea pigs and in the urine of hemoglobinuric dogs.

**Figure 3:**
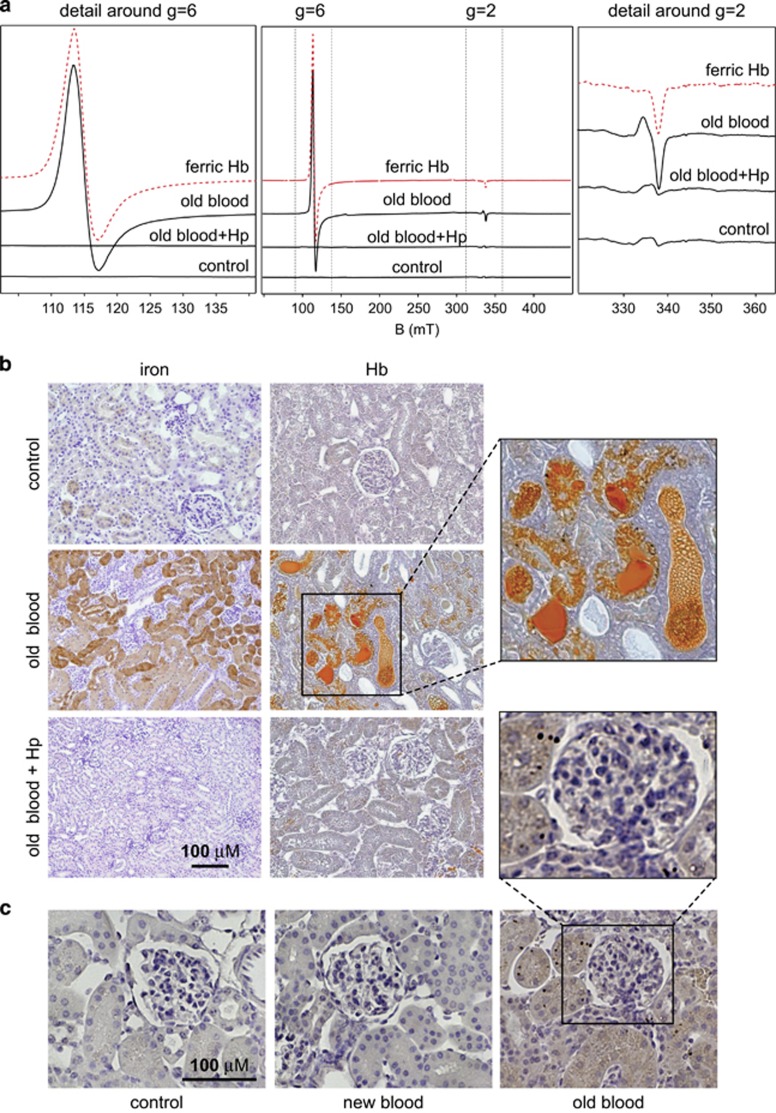
Renal hemoglobin accumulation and oxidative changes. (**a**) Electron paramagnetic resonance (EPR) spectroscopy of guinea pig kidney lysates from new blood-transfused, old blood-transfused, and old blood-transfused, Hp-treated animals. The spectra represent summed spectra of three samples from three individual animals measured at a 1 : 2 dilution of kidney lysates. The red spectrum indicates a ferric Hb(Fe^3+^) standard (200 *μ*M). The middle panel shows the whole recorded EPR spectrum. The right and left panels are zoomed regions around *g*=6 (ferric Hb signal) and *g*=2 (free radical signal). (**b**) Histochemistry for iron (left panels) and Okajima staining for Hb (right panels) of guinea pig renal tissues at 24 h after treatment. (**c**) Immunohistochemistry for 4-hydroxynonenal (4-HNE) at 24 h after treatment. The inset shows a higher resolution magnification of a glomerulus

Electron paramagnetic resonance (EPR) spectra of renal tissues from old blood-transfused animals were recorded at 6K and showed a strong signal around *g*=6, which is indicative of high-spin ferric Hb(Fe^3+^) (Figure 3a). No high-spin ferric Hb was present in the control tissues or renal tissues of old blood-transfused, Hp-treated guinea pigs. High-spin ferric Hb concentrations in renal tissues of old blood-transfused animals were 425±370 *μ*M (*n*=3). In this model of transfusion-related hemolysis, >95% of circulating, plasma-free Hb remained in the ferrous (Fe^2+^) state throughout the experiment, which indicates that the ferrous to ferric transformation must occur within the kidney tissue parenchyma and urinary filtrate. Figure 3b shows iron- and Hb-stained (Okajima staining) renal tissue sections. In old blood-transfused animals, strong Hb and iron deposition was observed in the tubule lumen and epithelial cells, respectively. Hp treatment prevented renal Hb exposure. The inset in Figure 3b shows that Hb predominantly accumulates in the tubule lumen and in proximal tubule epithelial cells.

Figure 3c shows the staining of renal tissues for 4-hydroxynonenal (4-HNE), which is a lipid peroxidation marker for oxidative damage to tissue proteins. Renal tissues from old blood-transfused animals exhibited 4-HNE-positive staining. In control, new blood-transfused, and old blood-transfused, Hp-treated animals, 4-HNE was not detected.

**Figure 4:**
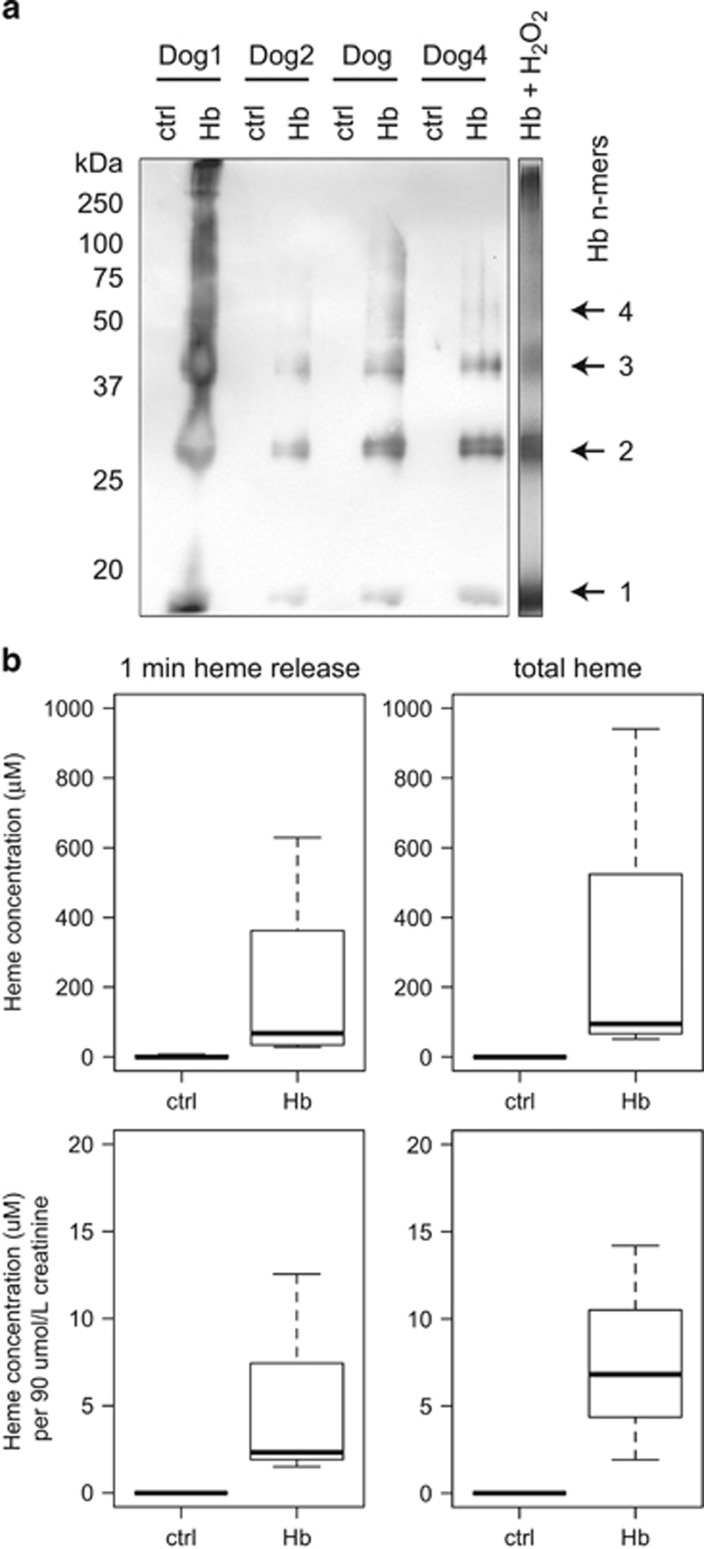
Globin cross-linking and free heme in urine of dogs with hemoglobinuria. (**a**) Hb-cross-linking products in dog urine. Urine samples of four dogs before (ctrl) and during infusion (Hb) were analyzed by western blotting. The most right lane is from an identical western blot that was performed with hydrogen peroxide (H_2_O_2_)-reacted purified Hb, which shows the typical peroxidation-induced Hb-cross-linking pattern. (**b**) Free heme concentrations in dog urine (*n*=8 Hb infused animals) were estimated with a hemopexin capture assay. Post-infusion urine samples were incubated with an excess of hemopexin for 1 min (1 min heme release) or 60 min (total heme). The concentration of heme–hemopexin was measured at the end of the incubation time by spectrophotometry. The lower panels indicate heme concentrations that were normalized to an estimated creatinine concentration of 90 *μ*M in the proximal tubule

As an alternative model of hemoglobinuria with a higher yield of urine volume we continuously infused adult beagle dogs with stroma-free ferrous Hb(Fe^2+^) for 6 h. Total Hb exposure resulted in a plasma AUC_0-∞_ of 4532±251 *μ*mol heme-h/l and a urinary Hb accumulation of 312.5±35 *μ*mol/l. To search for markers of intrarenal oxidative processes in this model, we analyzed the urine for Hb-cross-linking products, which represent the end product of oxidative Hb reactions.^20^ Figure 4a shows a reducing Hb-specific western blot from four representative dog urine samples in parallel with a hydrogen peroxide-treated Hb sample (hydrogen peroxide:Hb=10 : 1, 1 h reaction time at 37 °C). Covalent globin cross-linking products (dimer, trimer, tetramer, etc.) were detected in all dog urine samples during, but not before hemoglobinuria occurred.

We also quantified free heme in dog urine by using a hemopexin capture assay. Urine-free-heme concentrations were within the range of 40–600 *μ*M during hemoglobinuria. To estimate the range of free heme in tubule system these urine heme concentrations were normalized to a creatinine concentration of 90 *μ*M, which is the concentration that is expected in the primary glomerular filtrate. The normalized data shown in Figure 4b indicate that free heme accumulation in the renal tubule system may be within a range of 2–15 *μ*M in proximal segments to up to several hundred *μ*M in more distal tubule compartments.

### Free heme toxicity in human proximal tubule (HK-2) cells

**Figure 5:**
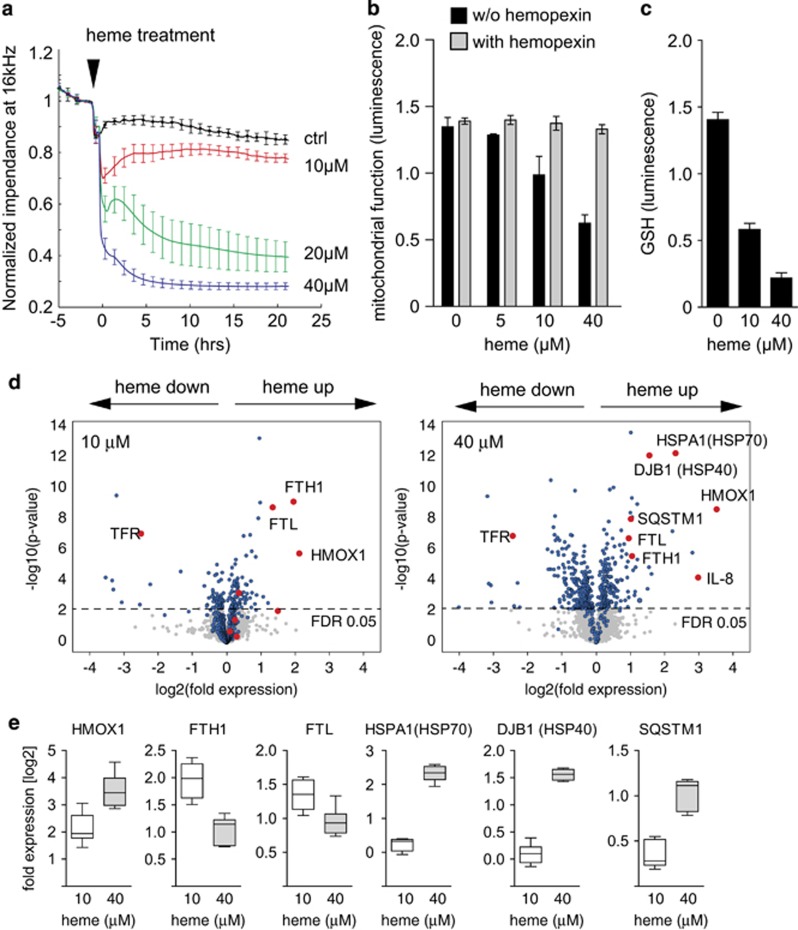
Free heme toxicity in renal epithelial cells. (**a**) Electrical monolayer resistance of confluent HK-2 renal epithelial cells was analyzed with an electric cell–substrate impedance sensing (ECIS) instrument before and after treatment with hemin (0 *μ*M, 10 *μ*M, 20 *μ*M, 40 *μ*M) in serum-free medium (*n*=4 biologic replicates per treatment). The arrowhead indicates the time point of hemin treatment. (**b**) Relative cellular ATP concentrations of HK-2 cells after treatment with heme (0–40 *μ*M) for 8 h (*n*=8 biologic replicates per treatment). Experiments were performed in serum-free medium in the absence or presence of hemopexin. (**c**) Relative reduced glutathione (GSH) concentrations in control and heme-treated (4 h) HK-2 cells (*n*=8 replicates per treatment). (**d**) Stable isotope labeling by amino acids in cell culture (SILAC)-based mass spectrometry analysis of the heme-induced proteome. The two volcano plots illustrate differential protein expression of HK-2 cells that were treated with 10 *μ*M heme (left plot) or 40 *μ*M heme (right plot) for 12 h. The *x* axis indicates relative log_2_ protein expression ratios of treated *versus* control cells. The data are representative of six biologic samples per condition. Significantly regulated proteins (in any condition) are highlighted in blue (FDR 5%). Only proteins that were detected in at least three of six samples per condition were included in this analysis. (**e**) Box plot representation of selected signature protein regulation (*n*=6 biologic replicas per condition)

Free heme, which is released from ferric Hb(Fe^3+^), was identified as a likely mediator of hemoglobinuria-associated damage. To estimate the potential toxicity of free heme in urine toward tubule cells and to validate the *in vivo* gene expression studies with an *in vitro* model, we treated HK-2 renal tubule epithelial cells with free heme under serum-free conditions. As shown in Figure 5a, heme concentrations of 10 *μ*M trigger an acute but transient decrease in epithelial electrical resistance, whereas concentrations >20 *μ*M cause an irreversible loss of barrier function. Accordingly, heme exposures of more than 10 *μ*M caused the significant and progressive depletion of cellular ATP, which was measured after an 8-h exposure period. This ATP depletion was prevented by the addition of the heme scavenger, hemopexin (Figure 5b). Reduced glutathione (GSH) was also depleted after 4 h of heme exposure, indicating that heme induces oxidative stress in exposed cells (Figure 5c). These data suggest that the estimated free heme concentrations that occur in the renal tubular system during severe intravascular hemolysis are in the range of heme concentrations that could trigger oxidative stress and cell damage to the renal epithelium.

### Quantitative proteome profiling identifies the response to unfolded proteins as a potential damage pathway in heme-exposed HK-2 cells

We incubated SILAC-labeled HK-2 cells with or without heme (10 and 40 *μ*M) for 8 h and defined heme-triggered proteome changes by LC-MS/MS. In six replicate experiments, we identified and relatively quantified 3108 proteins. The volcano plots in Figure 5d illustrate the relative protein expression changes of all proteins that were identified in at least three of the six analyzed biologic replicates. In agreement with the electrical cell–substrate impedance sensing data described above, the proteome changes triggered by 10 *μ*M heme were indicative of an adaptive response with prominent induction of HMOX1 and ferritin light (FTL) and heavy (FTH1) chains (Figure 5d, left panel). However, in the presence of 40 *μ*M heme, the toxic response was characterized by the strong induction of heat shock proteins, namely HSP70 (HSPA1B, HSPA4, HSPA5, DNAJB1), HSP72 (HSPA1A), HSP105 (HSPH1), and HSP10 (HSPE1), as well as the proteasome adaptor protein sequestosome. These proteins were upregulated in addition to the adaptive HMOX1 and ferritin (FTL, FTH1) (Figure 5d, right panel). Figure 5e shows box plots of the relative expression levels of selected signature proteins. Details of protein expression data are provided in the Supplementary data.

**Figure 6:**
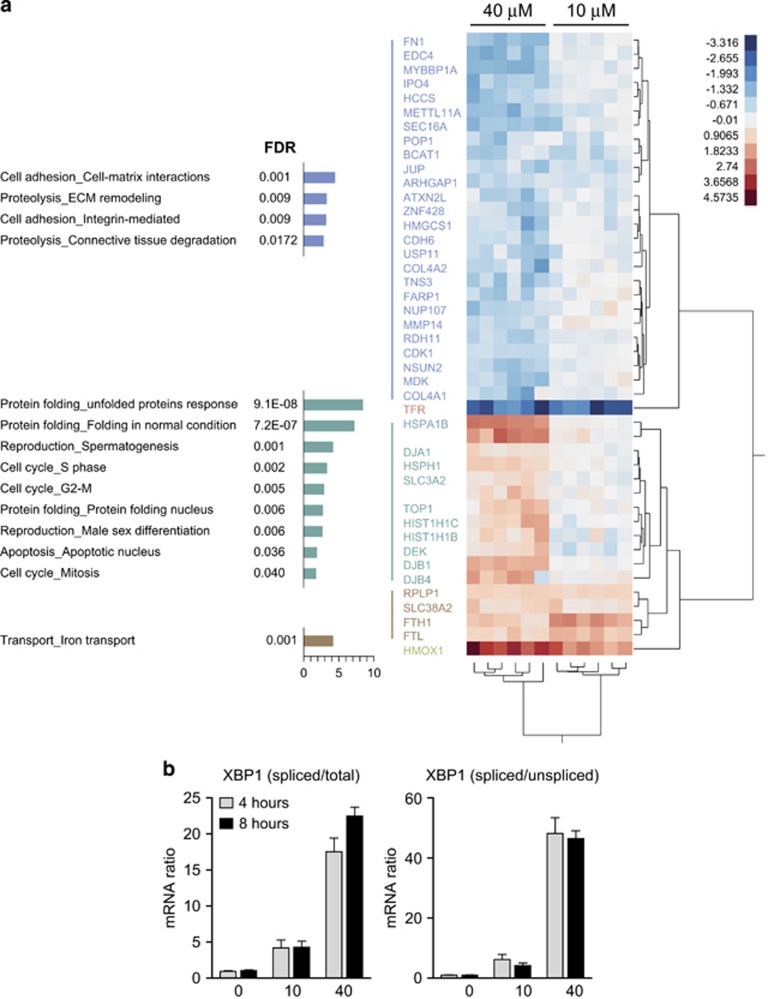
Free heme-triggered gene expression in renal epithelial HK-2 cells. (**a**) Hierarchical clustering analysis of all HK-2 proteins that were up (red) or down (blue) regulated by 10 or 40 *μ*M heme compared with control cells. Only proteins that were detected in all six samples per condition were included in this analysis. Three protein clusters were identified. The brown cluster was upregulated by both heme exposures, the green cluster was only upregulated at 40 *μ*M heme, and the blue cluster was downregulated by 40 *μ*M heme. In addition, the analysis identified the upregulation of HMOX1 and the suppression of the transferrin receptor (TFR) in all heme-treated samples. Significant results of a MetaCore analysis for the enrichment of functional process networks in the three different protein clusters are shown on the left. (**b**) X-box binding protein 1 (XBP1) splicing in heme-exposed (8 h) HK-2 cells was measured as a marker for the unfolded protein response

Figure 6a shows a hierarchical clustering analysis of all proteins that exhibit significantly different expression patterns between heme-treated and control cells (FDR 0.05, fold change >1.5). With this analysis, we could differentiate three principle clusters of proteins that were altered in expression by heme treatment. A small number of proteins (brown cluster) were induced by both heme concentrations. MetaCore analysis related these genes to iron transport. A second cluster of proteins (green cluster) identified genes with increased expression at 40 *μ*M. These ‘heme stress'-related proteins represent the response to unfolded proteins, as a potential pathway of cell damage. The third cluster identified a heme-suppressed group of proteins (blue cluster). This group represents biologic functions that are related to cell adhesion and proteolysis. In the presence of both 10 and 40 *μ*M, the transferrin receptor was identified as the most suppressed protein.

The heme-triggered activation of the UPR, which was suggested by the screening analysis, could be confirmed in HK-2 cells by the reverse transcription-polymerase chain reaction (PCR)-based splice assay for X-box binding protein 1 (XBP1).^21^ The ratio of spliced XBP1 to either total XBP1 or unspliced XBP1 was strongly increased by 40 *μ*M heme for 4 and 8 h. A minor increase in spliced XBP1 was observed with exposure to a sub-toxic concentration of heme (<10 *μ*M) (Figure 6b).

## Discussion

In this study, we systematically evaluated an animal model of hemoglobinuria in the guinea pig, dog, and a relevant *in vitro* cell culture model to characterize molecular pathways and underlying biochemical mechanisms that may lead to renal injury during hemolysis. The key observations were related to the sequence of intrarenal oxidative reactions and free heme-triggered toxicity, which may ultimately lead to tubular dysfunction and damage.

Ferrous Hb(Fe^2+^) is the principle iron transition state of free Hb that is found in the circulation of patients with hemolysis. It exhibits NO-scavenging activity, which can cause hypertension and ischemia. However, ferrous Hb is relatively inert regarding its potential toxic effect on cells and tissues. Therefore, it is possible that renal damage during hemoglobinuria may be caused by secondary reaction products of glomerular-filtered ferrous Hb(Fe^2+^). A candidate mediator of secondary Hb toxicity is free heme, which can be released when ferrous Hb(Fe^2+^) is oxidized to ferric Hb(Fe^3+^). Toxicity of free heme has been mainly attributed to its pro-oxidative nature, which may either directly damage cells or may promote generation of toxic lipid-oxidation products.^22, 23, 24, 25, 26, 27^ Several pathways of renal tubule cell death may be relevant to AKI and renal failure following sustained exposures to Hb and its components (globin, heme, and iron). Besides apoptosis, the two non-apoptotic cell death pathways necroptosis^28^ and ferroptosis^29, 30^ are of particular interest in response to heme-triggered oxidative stress. Iron-regulated ferroptosis has been shown to be an important cell death pathway in the kidney and upon cardiac ischemia and reperfusion injury.^29, 30^ In the kidney, ischemia followed by reperfusion has also been shown to induce ferroptosis in renal tubules though lipid peroxide accumulation. This process could be prevented by potent inhibitors of lipid peroxidation known as ferrostatins.^31^ We have examined the potential effects of specific pharmacologic inhibitors of apoptosis, necroptosis, and ferroptosis in our HK-2 cell culture heme toxicity model but could not show any significant effects, indicating that none of these pathways is exclusively active in heme-triggered cell death (data not shown).

We have recently shown that two principle and mutually interacting activities of heme can cause cellular damage if the intracellular levels of porphyrin can not be adequately controlled by heme oxygenases.^7^ At high intracellular concentrations, heme and other porphyrins can bind to high-affinity-binding sites within the 26S catalytic unit of the proteasome and inhibit its function.^7, 32^ This proteasome inhibitor function of heme is thought to impair cellular repair mechanisms, thus accelerating heme-induced oxidative injury. Ultimately, we found that uncontrolled cellular heme levels can activate the response to unfolded proteins and associated apoptosis pathways in mouse embryonic fibroblast cells. The UPR is a conserved response, which can be triggered by several extracellular insults, such as low nutrients, hypoxia, and inhibition of proteasome function. Such triggers can result in the accumulation of misfolded proteins in the ER, thereby causing ER stress and initiating the UPR.^33, 34^ The UPR increases the biosynthetic capacity and decreases the biosynthetic burden of the ER to maintain cellular homeostasis. However, when the stress cannot be compensated by the UPR, cellular apoptosis occurs.

The idea that severe hemolysis *in vivo* could trigger oxidative stress and the UPR in kidneys was reinforced in our studies by the analysis of the renal transcriptome of old blood-transfused guinea pigs. In a MetaCore analysis of the hemoglobinuria-associated transcriptome, we identified the same cellular processes that were also characteristic of the combined oxidative stress and proteasome inhibitor response of heme-exposed murine embryonic fibroblasts in our previous studies, as well as in the proteomic profile of heme-exposed HK-2 cells in the current study. In particular, the response to unfolded proteins appeared to be the most enriched process in all heme exposure models. The renal gene regulation data could be validated by the immunohistologic examination of HSP70 and HMOX1, which were both enhanced in the proximal tubule cells of old blood-transfused animals. The described changes were associated with an increase in the mRNA expression of renal NGAL, which is a relevant marker of renal tubular injury in AKI. The old blood transfusion-associated gene expression was not activated in animals that were either transfused with new blood or treated with the Hb scavenger, haptoglobin. This indicates that hemoglobinuria is the essential trigger of the observed tissue responses.

Other studies demonstrated that heme can trigger the activation of Toll-like receptor 4 and inflammasomes, thus leading to inflammatory reactions.^5, 35, 36, 37^ In our SAGE analysis of kidneys from old blood-transfused guinea pigs, a number of inflammation-associated gene products were upregulated and this was associated with sporadic macrophage accumulation in kidney (data not shown). None of these gene products was detected in new blood-transfused or old blood-transfused, Hp-treated animals. This pro-inflammatory response may represent either an unspecific tissue response to injury or it points to a specific activation of pro-inflammatory signaling by heme.

To substantiate the concept that oxidative Hb reactions with accumulation of ferric Hb(Fe^3+^) and free heme may lead to tubule damage during hemoglobinuria we have searched for markers of Hb-triggered oxidative processes in two *in vivo* models of hemoglobinuria. First, quantification of high-spin ferric Hb(Fe^3+^), which is the initial Hb oxidation product resulting from the oxidation of ferrous oxyHb(Fe^2+^), in the kidneys of the old blood-transfused guinea pigs suggests very high concentrations of ferric Hb can accumulate in renal tissues at 24 h after old blood transfusion. These EPR spectroscopy data refer to the total renal ferric Hb(Fe^3+^) concentration. However, the distribution of iron and Hb deposition was not homogeneous within the kidney, and the highest accumulation was detected in proximal tubule cells. This heterogeneity of renal Hb distribution may suggest exposures to considerably higher ferric Hb(Fe^3+^) concentrations (>800 *μ*M). Within ferric Hb(Fe^3+^), heme coordinates weakly, and ferric Hb(Fe^3+^) is therefore a more readily available source of heme. We also used a hemopexin capture assay in the urine of hemoglobinuric dogs and estimated that >10 *μ*M of heme are in a very loosely protein bound state and could be considered as free heme in renal tubular fluid. With the >400 *μ*M ferric Hb(Fe^3+^) concentration estimate that could be measured by EPR in the guinea pig renal cortex, we expect that significantly higher concentrations of free heme would occur locally during more severe renal Hb exposure.

In the absence of high-affinity scavenger proteins, such as hemopexin, free heme can transfer from ferric Hb(Fe^3+^) to lipid compartments, where it may accelerate peroxidative reactions. These reactions lead to the formation of lipid peroxides and toxic lipid-oxidation end products.^6^ The accumulation of 4-HNE adducts in the renal tissue of old blood-transfused guinea pigs suggests that such reactions do occur in the kidney during severe hemolysis. It has also been proposed that the peroxidase reaction of ferric Hb(Fe^3+^) with hydrogen peroxide or lipid peroxides could accelerate Hb toxicity through the generation of free radicals and the accumulation of globin oxidation products.^20, 38, 39, 40^ In this study, covalent globin cross-linking products were identified in the urine of our canine hemoglobinuria model. Such cross-linking products typically occur as a result of radical-generating Hb reactions, presumably as a result of dityrosine formation between radicalized α/β globin chains.

The above-discussed findings provide strong *in vivo* evidence that high concentrations of ferric Hb(Fe^3+^) and free heme can accumulate in the renal cortex during hemolysis. Subsequently, heme-triggered oxidative reactions may lead to tubular damage. We extrapolated these observations to an *in vitro* toxicity model to reinforce potential cellular damage pathways in a renal epithelial cell line. These studies suggest that the estimated *in vivo* free heme concentrations in tubules may trigger a range of cellular responses from adaptive to toxic. At the lower heme concentration tested (10 *μ*M), the response of human kidney HK-2 cells appeared to be adaptive with the strong induction of heme defense genes (HMOX1 and ferritin). This lower heme exposure resulted in a transient deregulation of epithelial barrier function, which recovered completely within a few hours. However, at higher heme concentrations (20 and 40 *μ*M), we observed a cytotoxic response with the irreversible breakdown of the epithelial barrier. Similar to our previous studies in HMOX1 knockout cells, we found that the toxic heme exposure of HK-2 renal epithelial cells caused oxidative cell stress with the depletion of cellular GSH and ATP and ultimately triggered the response to unfolded proteins.

In conclusion, our results suggest that during severe hemoglobinuria, oxidative processes do occur locally in the kidney. This promoted the accumulation of ferric Hb(Fe^3+^) and the release of free heme in the tubular system. Free heme triggered an oxidative stress response and the response to unfolded proteins in epithelial cells, which may ultimately lead to tubular dysfunction with AKI. The prevention of renal Hb filtration by Hp may be a therapeutic strategy to block renal Hb exposure and to rescue renal function in patients with severe hemoglobinuria.

## Materials and Methods

### Animal experiments

The US FDA/CBER Institutional Animal Care and Use Committee approved all animal studies. Surgical procedures, as well as the collection, storage, and transfusion of red blood cells, were performed, as described previously.^14^ Blood was transfused by an 80% volume-by-volume exchange system at a rate of 0.25 ml/min with new blood (stored for 2 days) or old blood (stored for 28 days). Some animals were treated with 750 mg Hp at the time of old blood transfusion. Untreated control animals underwent all surgical procedures without transfusion. Mixed-phenotype Hp from human plasma was obtained from CSL Behring (Bern, Switzerland). Anesthetized dogs were infused with stoma-free human ferrous Hb(Fe^2+^) to a target plasma concentrations of 150 *μ*M free heme for 6 h. Urine was collected by suprabubic puncture.

### SAGE

Total RNA was purified from tissue homogenates with the RNeasy Mini Kit (Qiagen, Hombrechtikon, Switzerland). An on-column DNA digestion step (RNase-Free DNase Set, Qiagen) was included. RNA integrity was checked with a Bioanalyzer 2100 instrument (Agilent Technologies, Basel, Switzerland), and only high-quality RNA (RNA integrity number >7.0) was used for further analyses. Total RNA (10 *μ*g) was used for library preparation with the SOLiD-SAGE Kit with Barcoding Adaptor Module (Applied Biosystems, Foster City, CA, USA). Library sequencing was performed with an AB SOLiD 5500xl system (Applied Biosystems) at the Functional Genomics Center Zürich in Switzerland.

SOLiD-SAGE tags were analyzed with the SOLiD-SAGE software (v.1.10; Applied Biosystems) by mapping the SAGE tags to the cavPor3 sequence database. The output was the copy number of each tag in a particular library. The mean normalized library size was 11.20 million, 11.86 million, 10.61 million, and 10.17 million reads for the new blood, old blood, old blood+Hp, and control guinea pig kidney cortex, respectively. For analysis, the tag length was set to 22 bases, and the maximum allowed mismatches was set to one. For data normalization and statistical tests to identify tags that originated from differentially expressed transcripts, we used the edgeR package (v.2.6.12; www.bioconductor.com). A transcript was considered to be differentially expressed if all of the following criteria were met: Log_2_ ratio >1, FDR level <0.05, and definite presence of the transcript (read count >50 in the samples of at least one group). Centroid clustering analyses of log-transformed ratio data and visualization were performed with JMP Genomics 5.0 (SAS, Böblingen, Germany). The treatment discriminative gene cluster consisting of 149 upregulated genes after old blood transfusion was analyzed with the MetaCore analysis software (Thomson Reuters, New York, NY, USA) for the enrichment of functional gene networks.

### EPR spectroscopy

For EPR analysis snap-frozen renal tissues were partially homogenized with a tissue homogenizer in cold phosphate-buffered saline (PBS) (v/v 1 : 1). The experiments were performed in a Bruker Elexsys spectrometer equipped with a resonator ER 4122 SHQ also from Bruker BioSpin (Fällanden, Switzerland). The temperature of the samples was 6 K, which was reached using a He gas-flow cryostat and a temperature controller ITC 503 from Oxford Instruments (Abingdon, UK). The microwave frequency was 9.46 GHz, the microwave power in the samples 0.13 mW and the magnetic field was modulated at 100 kHz with an amplitude of 1 mT. In addition to the renal tissue samples, two standard solutions of Hb of 100 and 200 *μ*M were measured and used to calculate the concentration of Hb in the samples. As the signals of Hb in tissue and solution were practically identical the concentration calculation was based on the intensity ratio of the spectra.

### Western blot analysis of the oxidative polymers of Hb

Dog urine containing 5 *μ*g Hb, as measured by spectrophotometry, was diluted 1 : 2 in Läemmli-Buffer containing 5% of 2-mercaptoethanol and boiled at 95 °C for 5 min. Proteins were separated on a Criterion Any-kD SDS-PAGE Gel (Bio-Rad, Cressier, Switzerland) and blotted on a polyvinylidene fluoride membrane with 0.4-*μ*m pores (Thermo Scientific, Reinach, Switzerland). After blocking in 10% goat serum, 10 g/l bovine albumin, and 0.1% Tween-20, membranes were incubated with a monoclonal mouse anti-Hb antibody (Abcam, #ab33844, Cambridge, UK) that was diluted 1 : 10 000 in 10% blocking buffer in PBS for 1 h, followed by incubation with a horseradish peroxidase-coupled goat anti-mouse IgG antibody (Amersham, GE Healthcare, Glattbrugg, Switzerland) at a dilution of 1 : 10 000 in 10% blocking buffer in PBS for 6 h. Hb was visualized by using the SuperSignal West Femto Maximum Sensitivity Substrate (Thermo Scientific) in a Chemidoc-XRS imager (Bio-Rad). For control samples, pure human HbA_1_ (10 *μ*M) was mixed with hydrogen peroxide in a 1 : 10 molar ratio at 37 °C for 60 min in PBS pH 7.4. Oxidized Hb (5 *μ*g) was then separated on a Criterion Any-kD Gel and developed using Coomassie Brilliant Blue staining. For alignment of the blot and SDS-PAGE gel, Precision Plus All Blue Protein Size Markers (Bio-Rad) were used.

### Non-heme iron and globin histochemistry

Kidney sections were incubated with Perls iron reagent containing 5% potassium ferrocyanide and 2% hydrochloric acid for 45 min at room temperature, after which they were rinsed in deionized water. Sections were then incubated with 0.3% hydrogen peroxide and 0.01 M sodium azide in methanol for 30 min at room temperature. All sections were rinsed in 0.1 M phosphate buffer, pH 7.4, and incubated with diaminobenzidine (SigmaFast DAB, Sigma) for 3 min. After the incubation, sections were washed in deionized water and lightly counterstained with Gill's II hematoxylin. All images were acquired using an Olympus IX71 inverted microscope equipped with an Olympus DP70 digital camera. Globin chain deposition (Okajima staining)—sections were incubated with 100 ml of 5% Alizarin Red and 50 ml of 10% phosphomolybdic acid for 1 h, washed in deionized water, and lightly counterstained with Gill's II hematoxylin.

### Immunohistochemistry and immunofluorescence

Tissue slides were de-paraffinized and hydrated in SafeClear and graded ethanol preparations. Antigen retrieval was performed in sodium citrate, and peroxidase inhibition was carried out in PBS containing 30% hydrogen peroxide and 0.05% Tween-20. Blocking was carried out by incubations with 10% horse serum and 0.25% Triton X-100 in PBS for 1 h at ambient temperature. 4-HNE was detected with a mouse monoclonal primary antibody (Clone HNEJ-2, Genox, Baltimore, MD, United States) and a biotinylated secondary antibody (anti-mouse IgG rat-adsorbed (BA2001, Vector Labs, Burlingame, CA, USA), which were visualized with avidin-biotin complex reagent (Vectastain, Vector labs) and developed with 250 *μ*l diaminobenzidine and hydrogen peroxide (Sigma-Aldrich, Buchs, Switzerland) for 3 min. Counterstaining was performed in hematoxylin for 15 s. Slides were viewed, and images were captured under a light microscope at × 60 magnification. Optimization was carried out by adjusting the brightness and contrast of all images equally using Adobe Photoshop (Adobe, Adobe Systems GmbH, Zürich, Switzerland). For immunofluorescence, the following primary antibodies were used: mouse monoclonal antibody (3A3) against HSP70 (Abcam, ab5439) and rabbit polyclonal antibody against HMOX1 (Enzo SPA-895, Lausen, Switzerland). Alexa 568-conjugated secondary antibodies were used (Invitrogen, Thermo Scientific, Reinach, Switzerland). Images were acquired with a Zeiss Axioscope 2 fluorescence microscope (Carl Zeiss AG, Feldbach, Austria) with 200-fold optical magnification. Equal image acquisition settings were used for all images. Channels were merged with ImageJ software (version 1.48c). Hb chain deposition (Okajima staining)—sections were incubated with 100 ml 5% Alizarin Red+50 ml 10% Phosphomolybdic acid for 1 h, washed in deionized water, and lightly counterstained with Gill's II hematoxylin.

### Heme/hemopexin capture assay

Because heme, ferric Hb(Fe^3+^), and hemichrome species extensively aggregate in urine, the direct quantification of free heme by spectrophotometry in these samples is difficult. In contrast, hemopexin specifically captures monomeric free heme. The heme–hemopexin complex yields a clear and specific visible spectrum that is proportional in intensity to the concentrations of free heme and heme that is loosely attached to proteins. Using PBS (pH 7.45), dog urine samples were diluted to a final Hb concentration of 10 *μ*M. Control urine samples containing no Hb were diluted by the same factor as their corresponding treated urine samples. All samples were pre-warmed to 37 °C. Hemopexin (CSL Behring) was added to a final concentration of 20 *μ*M, and absorbance spectra were recorded during the interval of 350–650 nm at 2-nm intervals (25-ms integration time) every 3 min for 1 h, starting 1 min after the addition of hemopexin using an Agilent Cary 60 spectrophotometer and a Varian PCB 1500 cuvette heater. The resulting spectra were quantified for methemoglobin, oxyhemoglobin, and heme–hemopexin using the non-negative least squares algorithm and the R statistics package, as described previously.^41^

### ECIS and the measurement of cellular ATP and GSH

ECIS was performed by growing HK-2 cells to confluence on 10E+ ECIS wells (Applied BioPhysics, Troy, NY, USA) in 1 : 1 DMEM:Ham's F-12 (Gibco), which was supplemented with 10% fetal calf serum (FCS), 0.05% ITS-A (Gibco), 100 *μ*M cortisol (Sigma-Aldrich), and penicillin-streptomycin (Gibco). ECIS resistance, impedance, and capacitance were recorded over the full spectrum for all wells. After equilibration, the medium was replaced with serum-free medium for 1 h, and then heme was added to the cells. The relative concentrations of ATP and GSH were measured by specific chemiluminescence assays (Promega).

### HK-2 SILAC Experiments

HK-2 cells were grown to confluence in 1 : 1 DMEM:Ham's F-12 medium that was deficient for lysine and arginine and supplemented with 10% dialyzed FCS, 0.05% ITS-A (Gibco, Thermo Scientific, Reinach, Switzerland), and 100 *μ*M cortisol (Sigma-Aldrich). Cells were then labeled with stable isotope-tagged arginine and lysine and cultivated for 24 h in serum-free medium before treatments with 0 (control), 10, and 40 *μ*M heme. Control samples were always labeled with light arginine and lysine, whereas the two heme-exposed samples were labeled with either medium or heavy amino acids. All samples were lysed with radioimmunoprecipitation assay buffer containing the Roche complete protease inhibitor cocktail and normalized for protein concentration (BCA-Assay, Pierce, Thermo Scientific, Reinach, Switzerland). Equal amounts of light, medium, and heavy labeled lysates were pooled before further processing. Proteins were reduced by 10 mM dithiotreitol, carboamidomethylated by 100 mM iodoacetamide, and digested using 1 *μ*g trypsin (Roche, Basel, Switzerland) per sample at room temperature overnight. Afterwards, they were cleaned up using ZipTips (Millipore, Merck, Schaffhausen, Switzerland) and analyzed using an Orbitrap Velos Mass Spectrometer (Thermo Scientific). Data were analyzed using MaxQuant.^42^ Settings are noted in the Supplementary data. The MaxQuant peptide output was pooled by proteins (if identified by at least a razor peptide), which were weighted by the total intensity of the detected ion. For statistical analysis (ANOVA, hierarchical clustering) and generation of plots we used JMP Genomics 6.0 (SAS Institute Inc.).

### Quantitative real-time PCR

Total RNA was isolated from guinea pig kidneys, and complementary DNA was synthesized. Real-time PCR reactions were performed using the Applied Biosystems ViiA 7 Real-Time PCR System and its TagMan Fast Universal PCR Master Mix (Applied Biosystems), according to the manufacturer's protocol. The custom-designed TaqMan probes and primer sets for guinea pig lipocalin 2 (forward: 5′-GTCCCACCACTGAGCAAGAT-3′, reverse: 5′-GTCATCTTTCAGCCCGTAGG-3′, TaqMan probe: 5′-CAAGACAAGTTCCAGGGGAA-3′) and glyceraldehyde 3-phosphate dehydrogenase (GAPDH; Cp03755743_g1) were purchased from Applied Biosystems. Real-time PCR for each gene was performed in triplicate and normalized to GAPDH values. Results are presented as fold change and expressed as means±S.E.M. Statistical analysis on real-time PCR was performed on raw Δ*C*_t_ data (*n*=4 animals per group).

### XBP1 splice assay

Complementary DNA was synthesized and real-time PCR reactions were performed by using the Fast SYBR Green Master Mix (Applied Biosystems) according to the manufacturer's protocol on a 7500 fast real-time PCR system (Applied Biosystems). Primers for spliced XBP1 (5′-CTGAGTCCGAATCAGGTGCAG-3′ and 5′-ATCCATGGGGAGATGTTC-3′), unspliced XBP1 (5′-CACACTCAGACTACGTGCA-3′ and 5′-ATCCATGGGGAGATGTTCTGG-3′) and total XBP1 (5′-TGGCCGGGTCTGCTGAGTCCG-3′ and 5′-ATCCATGGGGAGATGTTCTGG-3′) were designed according to Oslowski *et al.*^43^

## Abbreviations

4-HNE: 4-hydroxynonenal
AKI: acute kidney injury
ATP: adenosine triphosphate
AUC: area under the curve
EPR: electron paramagnetic resonance
ER: endoplasmatic reticulum
FCS: fetal calf serum
FDR: false discovery rate
FTH1: ferritin heavy chain 1
FTL: ferritin light chain
GSH: reduced gluthatione
Hb: hemoglobin
Hb(Fe2+): ferrous hemoglobin
Hb(Fe3+): ferric hemoglobin = met-hemoglobin
HMOX1: heme oxygenase 1
HSP70: heat shock protein 70
LC-MSMS: liquid chromatography coupled to mass spectrometry x 2
NGAL: neutrophil gelatinase associated lipocalin
NO: nitric oxide
PCR: polymerase chain reaction
SAGE: serial analysis of gene expression
SILAC: stable isotope labeling with amino acids in cell culture
UPR: unfolded protein response
XBP1: x-box binding protein 1
